# Transmission Lines in Capacitance Measurement Systems: An Investigation of Receiver Structures

**DOI:** 10.3390/s23031148

**Published:** 2023-01-19

**Authors:** Matthias Flatscher, Markus Neumayer, Thomas Bretterklieber, Hannes Wegleiter

**Affiliations:** 1 Institute of Electrical Measurement and Sensor Systems, Graz University of Technology, Inffeldgasse 33, 8010 Graz, Austria; 2 Christian Doppler Laboratory for Measurement Systems for Harsh Operating Conditions, Institute of Electrical Measurement and Sensor Systems, Graz University of Technology, Inffeldgasse 33, 8010 Graz, Austria

**Keywords:** frequency-spectroscopy, transmission line, noise, SNR, radio frequency, receiver circuit, impedance transformation

## Abstract

Dielectric sensing based on capacitive measurement technology is a favourable measurement approach in many industries and fields of application. From an electrical point of view, a coupling capacitance must be measured in the presence of stray capacitances. Different receiver circuit structures have been proposed for the underlying displacement current measurement. Ideally, the sensor assembly is directly connected to the sensor circuitry to minimize the influence with respect to these parasitic capacitances. However, under harsh operating conditions, e.g., at high temperatures, the sensor and the receiver circuit must be separated in order to protect the electronics. Consequently, the receiver circuit and the sensor have to be connected by cables, e.g., coaxial cables. The measurement setup differs significantly from the ideal design with a direct connection. In this paper, we investigate the behaviour of three common measurement circuits for capacitive measurements in instrumentations with cables. We study the interaction between the sensor and the electronics and analyse the operating behaviour of the circuit, as well as the operating states of the amplifiers used. We also address cross-sensitivities in the sensor design due to stray capacitances. The analyses are carried out for different cable lengths and measuring frequencies, and conditions for the usability of the circuit are deduced. In addition to the operational behaviour, we also evaluate the circuits by means of a noise analyses. Based on this analysis, we show a direct comparison of the circuits. The analysis is based on simulation studies, as well as collaborative measurements on test circuits where all circuit parameters are provided. The test circuits are realized with dedicated state-of-the-art circuit elements and, together with the analysis approach and the results, thus provide a basis for future developments.

## 1. Introduction

The characterisation and monitoring of materials and substance properties based on dielectric sensing is a well-established approach for many scientific and industrial applications [1]. Examples can be found in agricultural applications [2,3], monitoring of food [4], forestry [2,3,4,5,6,7], biomass [6], and biofuels [7]. For example, moisture content is an essential parameter for the quality of these goods. Due to its sensitivity to moisture, dielectric sensor technology is suitable for these measurements.

Yet the sensing capabilities of dielectric sensing have also been applied in various other applications; e.g., in [8] dielectric sensing is presented for ice sensing in environmental monitoring [9,10,11]. The capabilities also extend towards process tomography [12,13,14,15,16,17].

**Figure 1:**
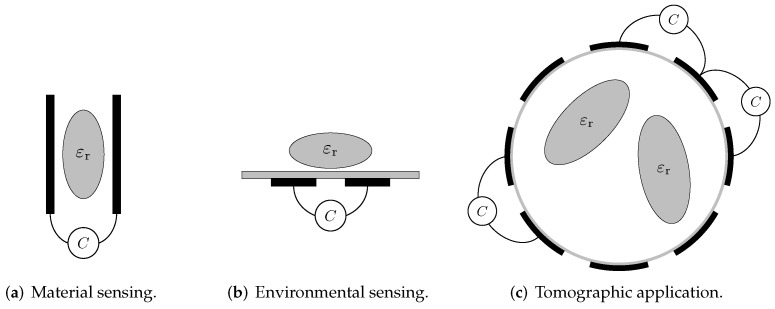
Illustration of different sensor schemes for dielectric sensing. The fundamental electrical measurement task is the determination of the coupling capacitances between electrodes.

Figure 1 gives an overview of different sensor schemes for the various applications of dielectric sensing. The black lines mark the electrodes. The ellipse-shaped object shows the material/sample to be characterised. The capacitance measurements are indicated by the instruments. Figure 1a depicts a probe scheme for material measurements [18]. Here, the material to be characterised is placed within a well-defined electrode array. Figure 1b shows a sensor arrangement for environmental measurements, where the material is placed in front of electrodes [19]. Figure 1c shows a sensor arrangement for process tomography where the material is inside a tube and the electrodes are placed outside.

In all sensor schemes shown in Figure 1, the capacitance meters are directly connected to the electrodes. This represents an ideal configuration for measuring capacitance [20]. However, in harsh environments, e.g., applications with high temperature exposure, this is often not possible. The electronics must be separated from the front end to reduce the stress and protect the electronics. As a rule of thumb, an increase in operating temperature of about 10 °C will result in a reduction in component life by half [21,22,23]. Conversely, a temperature reduction of about 10 °C leads to a doubling of the expected lifetime [23,24,25].

**Figure 2:**
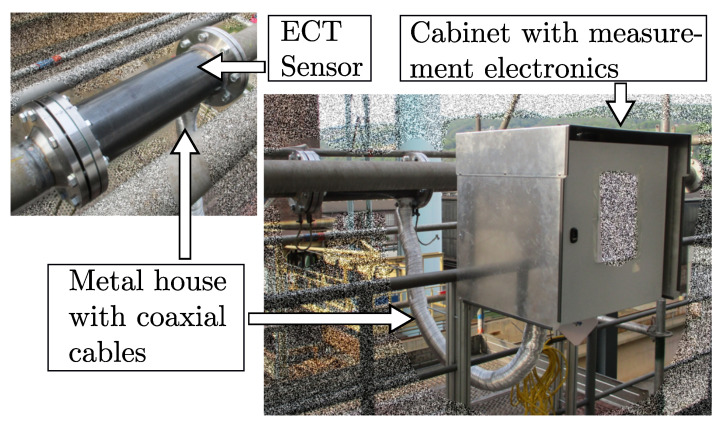
Photography of an ECT system in an industrial process plant. The measuring electronics is located in a cabinet, and the sensor electrodes and the electronics are connected via coaxial cables.

The subsequent separation of front-end and electronics requires suitable wiring of the elements, e.g., by coaxial cables [26,27,28]. Figure 2 shows an example of an ECT sensor in an industrial plant. The measuring electronics are located in a cabinet, and the sensor electrodes and the electronics are connected via coaxial cables. The length of the cables is in the range of 2.5m.

**Figure 3:**
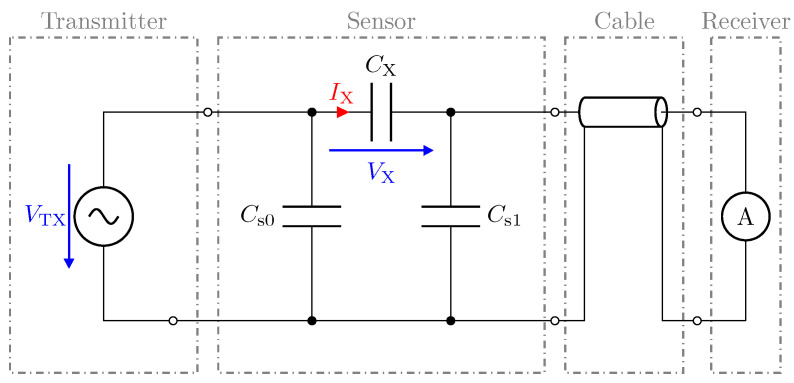
Simplified circuit representation of a capacitive measurement device. For low-Z measurements, a current measurement is used [26].

With regard to the properties for capacitive measurement, the influence of the cables on the measurement system must be investigated. Figure 3 shows a simplified capacitive measurement circuit, where $C_{X}$ represents the capacitance of interest, i.e., the capacitance between the electrodes. The capacitances $C_{s0}$ and $C_{s1}$ represent the stray capacitances of the transmitting electrode and the receiving electrode, respectively, with respect to the system ground (GND). Depending on the application, the stray capacitances can reach large values with respect to the interelectrode capacitance $C_{X}$. In electrical capacitance tomography (ECT), for example, the typical values for the interelectrode capacitance are in the range of some fF up to some pF [29,30]. To measure $C_{X}$, a transmitter (TX) provides a sinusoidal excitation signal of amplitude $V_{\mathrm{TX}}$ and frequency $f_{\mathrm{TX}}$, resulting in the displacement current $I_{\mathrm{TX}}$, which must be measured by a suitable receiver. This is illustrated by the amperemeter shown in Figure 3, which is connected to the sensor via the cable.

For the measurement of the displacement current $I_{X}$, different front-end structures have been proposed:

- A low-input impedance circuit based on a current to voltage converter [20]. This configuration is optimal for a direct connection of the sensor and circuitry, as it shunts parasitic capacitances. We refer to this as a low-Z receiver in this work.
- In [30], a resonant measurement circuit was proposed. This approach provides low input impedance and additional amplification due to a resonance. We refer to this as LCR receiver.
- An impedance-matched front-end design is proposed in [31], in which the input impedance of the receiver is matched to the wave impedance of the line. We refer to this as matched receiver.

In this article, we examine these three different circuit setups in terms of their measurement behaviour and noise performance. This also includes the sensitivity to stray capacitances. Thus, the analysis also extends the work presented in [31] with regard to these aspects. The analysis is carried out using measurements as well as collaborative simulation studies for different cable lengths for the three receiver structures. For this purpose, test circuits are built with dedicated state-of-the-art circuit elements. The behaviour of all systems is compared with a directly connected variant, i.e., without cables. The nature of the effects and mutual interactions requires a holistic analysis of the measurement system. Therefore, in addition to the comparative results for the different circuits, the research and modelling strategies are also new contributions presented with the article. The contribution of the work can therefore be summarised as follows.

- Holistic system analysis by considering the interaction between the sensor and electronics.
- Investigation of a directly attached sensor and electronics assembly as well as a spatial dislocated arrangement by means of the use of transmission lines.
- Simulation-based assessments of the achievable signal-to-noise ratio (SNR) and quantitative comparison of the topologies, considering the environmental impacts.
- Suggestion of established receiver structures and investigation of their applicability for spectroscopic applications.

Furthermore, for the test circuits, all circuit elements are stated, providing researchers a quantified basis for further research.

This paper is structured as follows. In Section 2, the different circuits are introduced and their basic behaviour for a direct connection, i.e., a measurement without cable, is discussed. In the Section 3, Section 4 and Section 5 the presented circuits are analysed when using coaxial lines for the sensor connection. The analysis is carried out using the test circuits and includes an analysis of the system behaviour and a noise analysis. Based on the individual results, a comparative summary is given in Section 6. Aspects such as sensitivity to stray capacitances, technical effort, and complexity are also addressed.

## 2. Overview of Possible Receiver Structures

In this section, we present the different receiver concepts, which are investigated and address their fundamental behaviour. We then outline the further research approach addressed in the Section 3, Section 4 and Section 5.

**Figure 4:**
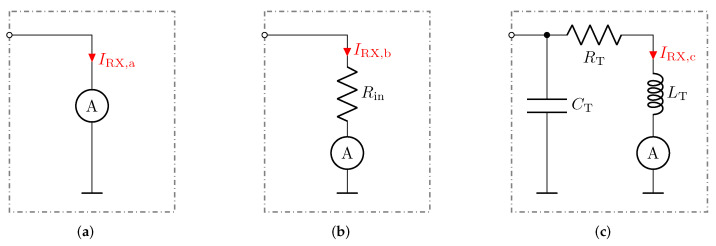
Illustration of three received current measurement circuits, applicable for the circuit shown in Figure 3. (**a**) Low-Z measurement circuit with an idealized input resistance equal to zero. (**b**) Matched measurement circuit, by means of maintaining a desired input resistance $R_{\mathrm{in}}$. (**c**) LCR measurement circuit. The resonance circuitry also provides filter characteristics.

Figure 4 depicts three receiver structures, which are considered in this work. They are referred to as low-Z measurement circuit, matched measurement circuit [31], and LCR measurement circuit [30], respectively. The receiver circuits were investigated with respect to their behaviour to measure the capacitance $C_{X}$ within the configuration depicted in Figure 3. The analysis carried out for typical capacitance values as they appear in electrical capacitance tomography, where stray capacitances are typically large compared to the inter-electrode capacitance [29,30]. An inter-electrode capacitance $C_{X}$ of 1 pF and a capacitance of 10 pF is used for the stray capacitances $C_{s0}$ and $C_{s1}$ [32].

### 2.1. Low-Z and Matched Receiver 

Figure 4a shows the idealized low-impedance implementation of the receiver structure, which maintains an input resistance equal to zero. Consequently, the stray capacitance $C_{s1}$ as shown in Figure 3 is short-circuited to the ground. Thus, the current ${\underline{I}}_{X}$ can be expressed as

(1) $${\underline{I}}_{X}=i\;\cdot \;2\;\cdot \;\pi \;\cdot \;f_{\mathrm{TX}}\;\cdot \;C_{X}\;\cdot \;{\underline{V}}_{\mathrm{TX}}$$

and the received current can be expressed by

(2) $${\underline{I}}_{\mathrm{RX},a}={\underline{I}}_{X}.$$

**Figure 5:**
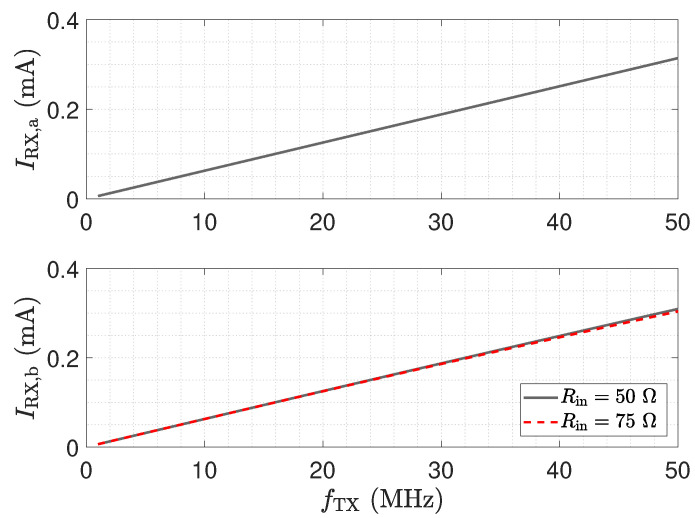
Receive current frequency responses for the low-Z circuit (upper plot) and matched input stage (lower plot) depicted in Figure 4a,b, respectively. An excitation voltage $V_{\mathrm{TX}}$ of 1 V is applied, $C_{X}=1\;pF$, $C_{s0}=C_{s1}=10\;pF$. The maximum low-Z received current at a frequency of 50 MHz is about 0.314 mA. The maximum received current emerging for the matched input stage at a frequency of 50 MHz is about 0.310 mA and 0.304 mA for $R_{\mathrm{in}}$ = 50 Ω and $R_{\mathrm{in}}$ = 75 Ω, respectively.

The corresponding frequency response is shown in the upper plot of Figure 5. At a signal frequency of 50 MHz, the received current is about 314 μA.

Figure 4b shows a displacement-current measurement circuit, maintaining a certain input resistance $R_{\mathrm{in}}$. For example, to be operated with transmission lines, $R_{\mathrm{in}}$ meets the characteristic wave impedance of the transmission line. The circuit shown in Figure 4b is referred to as z matched structure within this work. By considering the input resistance, the received current for the circuit shown in Figure 4b can be determined by

(3) $${\underline{I}}_{\mathrm{RX},b}=\frac{\frac{1}{i\;\cdot \;2\;\cdot \;\pi \;\cdot \;f_{\mathrm{TX}}\;\cdot \;C_{s1}}}{R_{\mathrm{in}}+\frac{1}{i\;\cdot \;2\;\cdot \;\pi \;\cdot \;f_{\mathrm{TX}}\;\cdot \;C_{s1}}}\;\cdot \;{\underline{I}}_{X,b}.$$

${\underline{I}}_{X,b}$ is the displacement current across $C_{X}$ caused by the resulting series impedance of $C_{X}$ and $C_{s1}\left|\right|R_{\mathrm{in}}$. The lower plot in Figure 5 shows the accompanying frequency responses of the current $I_{\mathrm{RX},b}$ for two input resistance values, meeting two typical transmission line impedances. The input resistor $R_{\mathrm{in}}$ creates a current divider with the stray capacitance of the receiver electrode $C_{s1}$, which leads to a decrease in the received current $I_{\mathrm{RX},b}$ for an increasing measurement frequency $f_{\mathrm{TX}}$. The maximum received current at a frequency of 50 MHz is about 0.31 mA and 0.304 mA for an input resistance of 50 Ω and 75 Ω, respectively.

**Figure 6:**
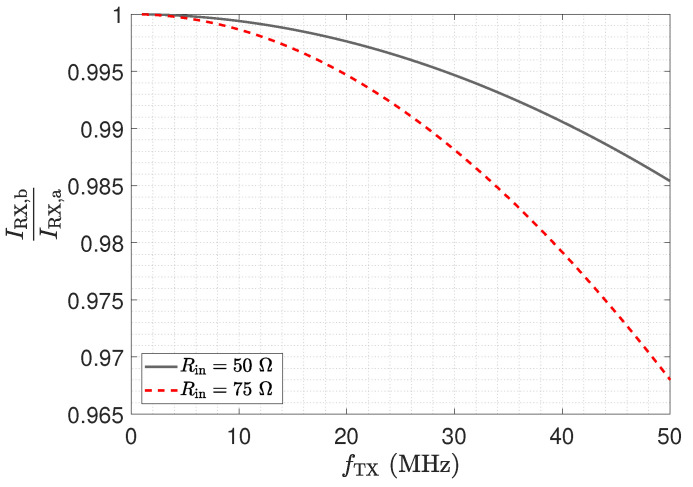
Current ratio $I_{\mathrm{RX},b}/I_{\mathrm{RX},a}$ of the received currents determined with the circuit topologies shown in Figure 4a,b.

Figure 6 shows the ratio of the matched received current to the low-Z received current $I_{\mathrm{RX},b}/I_{\mathrm{RX},a}$. The frequency response in Figure 6 highlights the impact of the stray capacitance $C_{s1}$ and the input resistance value on the determined received current magnitude. As can be seen, an increased input resistance value leads to a reduced received current at high frequencies for the matched structure. An input resistance of 50 Ω leads to a current reduction of about 1.5%. This result indicates a minor influence of $R_{\mathrm{in}}$ on the received current in the investigated frequency span, for a receive electrode stray capacitance $C_{s1}$ of 10 pF.

### 2.2. LCR Receiver

The circuitry in Figure 4c is referred to as resonant LCR receiver and has been suggested by various authors, as it provides improved electromagnetic compatibility (EMC) and additional amplification when operated at resonance frequency [33,34,35,36].

The impedance of a resonant inductor–capacitor resistor (LCR) parallel circuit shows a maximum when operated at its resonant frequency [37]. Intrinsic losses of the inductor $L_{T}$, represented by $R_{T}$, lead to a significantly lower input impedance [38]. Thus, the circuit falls also into the class of low-Z receiver structures [30,36], yet this has not to be confused with the low-Z circuit depicted in Figure 4a. The corresponding received current can be determined by

(4) $${\underline{I}}_{\mathrm{RX},c}=\frac{\frac{1}{i2\pi f_{\mathrm{TX}}\;\cdot \;\left(C_{s1}+C_{T}\right)}}{R_{T}+i2\pi f_{\mathrm{TX}}\;\cdot \;L_{T}+\frac{1}{i2\pi f_{\mathrm{TX}}\;\cdot \;\left(C_{s1}+C_{T}\right)}}\;\cdot \;{\underline{I}}_{X,c}.$$

**Figure 7:**
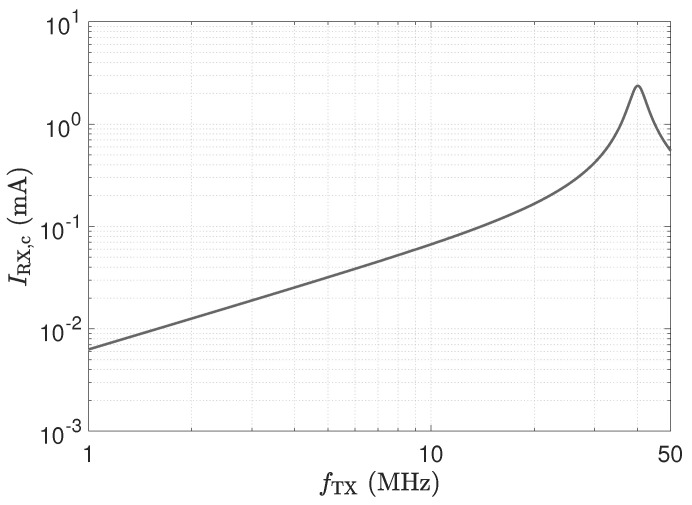
Frequency response of the current for the LCR input stage shown in Figure 4c. Component values stated in [30] were used to obtain a resonant frequency of 40 MHz. The LCR circuit has a quality factor *Q* of 9.6. An excitation voltage $V_{\mathrm{TX}}$ of 1 V is applied, $C_{X}=1\;pF$, $C_{s0}=C_{s1}=10\;pF$.

${\underline{I}}_{X,c}$ is the displacement current across $C_{X}$ caused by the resulting series impedance of $C_{X}$ and $(C_{s1}\left|\right|C_{T})||\left(L_{T}+R_{T}\right)$. In contrast to the other two receivers, this circuit requires a more careful setting of the components in the front-end. From a system point of view, the resonance frequency of the circuit has to be selected. In this work, we take the values for $L_{T}$, $C_{T}$ and $R_{T}$, as in [30], which leads to a resonance frequency of 40 MHz. This value was selected as frequencies in the range of a few 10 MHz allow for easy realisation [30]. Figure 7 depicts the corresponding frequency response of the current.

By utilizing the resonant circuitry to measure the displacement current in Figure 3, the parasitic stray capacitance $C_{s1}$ contributes to the resulting resonant frequency, which can be determined by [26]

(5) $$f_{0}=\frac{1}{2\;\cdot \;\pi \;\cdot \;\sqrt{L_{T}\;\cdot \;\left(C_{T}+C_{s1}\right)}}.$$

To minimize the undesired impact of parasitic capacitances, the implementation of $C_{T}$ by means of a tunable varactor diode is suggested [26,30]. The diode capacitance has to be adjustable over the expected stray capacitance range. Based on its principle, the LCR input stage provides a narrow frequency band around its resonance frequency, thus limiting this circuit to narrow-band spectroscopy applications. A resonance frequency tuning of about ±25% can be achieved by the use of a varactor diode [39]. The use of a switchable capacitor bank using digitally controlled switches would be a further possibility to increase the tunable frequency range. The used switches also introduce parasitic capacitances. Further, the switch-on resistance causes a reduction in the current gain, as the on-resistance of a closed switch leads to a reduction in the quality factor *Q* of the LCR circuit [26].

**Figure 8:**
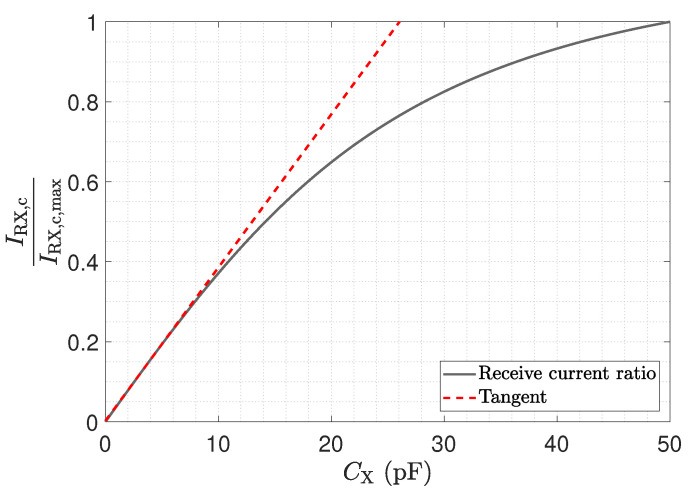
Normalized received current of the LCR circuit shown in Figure 4c for an excitation frequency of 40 MHz, as a function of the inter-electrode capacitance $C_{X}$. The tangent indicates the ideal linear behaviour.

As discussed, the impedance of a resonant LCR parallel circuit shows a maximum when operated at its resonant frequency, thus having an impact on the receiver circuit’s linearity [26]. Figure 8 shows the normalized received current for an excitation frequency of 40 MHz as a function of $C_{X}$. The red-dashed tangent shows the ideal linear behaviour. As can be seen, the investigated LCR structure shows an almost linear characteristic for capacitances up to 10 pF, which covers the range of typical inter-electrode capacitances occurring in ECT applications [29,30].

By comparing the analytical results in Figure 5 and Figure 6, and considering the influence of stray capacitance $C_{s1}$ on the resonant frequency of the LCR circuit, shown in Equation (5), the direct attached assembly of the sensor and the low-Z receiver structure exhibits superior immunity to stray capacitances. Therefore, a direct attached configuration of the low-Z circuit has been suggested by various authors [40,41,42]. The low-impedance input stage is also used by a variety of measurement systems in combination with cables [43,44,45,46].

### 2.3. Outline of the Further Analysis

**Figure 9:**
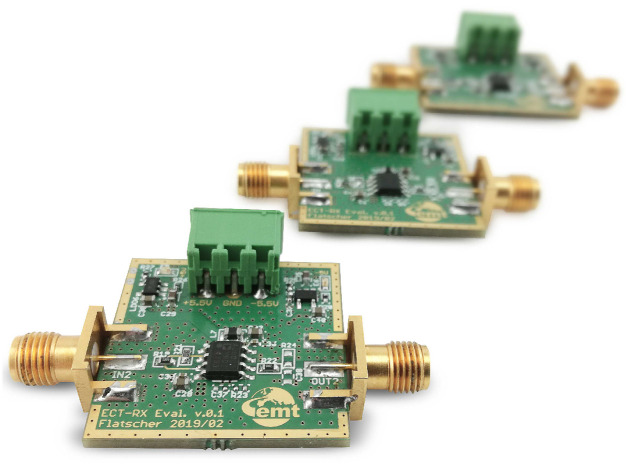
Realized circuits of the theoretical representations in Figure 4 to perform comparative measurements.

Given the initial discussion of the three different receiver structures, Section 3, Section 4 and Section 5 present a technical analysis of the circuits regarding their properties within instrumentations, which include transmission lines. Each section is divided into two parts. The first part of the analysis addresses the aspects about the electrical behaviour of the circuit. The second part shows a noise analysis of the circuit. For each of the proposed receiver structures a test board was built, using selected circuit elements, e.g., dedicated high speed opamps. Figure 9 shows photographs of the circuit boards. Details about the actual realization are addressed in the specific Sections.

## 3. Low-Z Receiver

**Figure 10:**
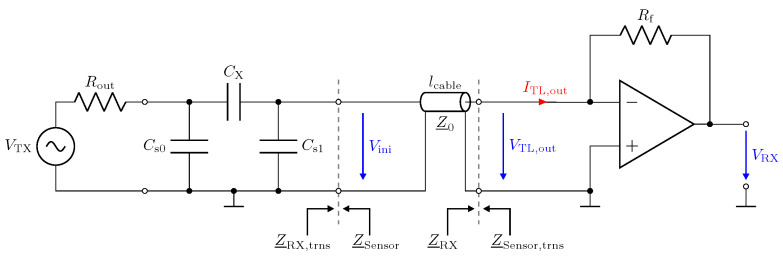
Low-impedance receiver circuit connected to a capacitive Π-network by means of a transmission line with length $l_{\mathrm{cable}}$ and impedance ${\underline{Z}}_{0}=50\;\Omega$. $R_{\mathrm{out}}=50\;\Omega$, $R_{f}=500\;\Omega$, $C_{X}=1\;pF$, $C_{s0}=C_{s1}=10\;pF$.

In this section, we address the behaviour of a low-Z receiver in combination with transmission lines. Figure 10 shows the circuit realization of the low-Z input stage by means of a transimpedance amplifier (TIA). The circuitry uses the AD8000 opamp from Analog Devices [47]. It provides a low-impedance virtual-ground [48] for the investigated frequency span [31,32]. The capacitive Π-network shown in Figure 10 has been realized by lumped capacitors. All component values are listed in the caption.

The impedances ${\underline{Z}}_{\mathrm{RX}}$ and ${\underline{Z}}_{\mathrm{Sensor}}$ in Figure 10 denote the impedances of the circuitry and the sensor. ${\underline{Z}}_{\mathrm{RX},\mathrm{trns}}$ and ${\underline{Z}}_{\mathrm{Sensor},\mathrm{trns}}$ are the corresponding impedances measured with the transmission line.

### 3.1. Low-Z Receiver: Behaviour of ${\underline{Z}}_{\mathrm{RX},\mathrm{trns}}$ and ${\underline{Z}}_{\mathrm{Sensor},\mathrm{trns}}$

To study the influence of the transmission lines, network analyser [49] measurements were used to investigate the low-Z receiver performance. The network analyser was calibrated by means of a through-open-short-match (TOSM) calibration [50]. The transmission line has a characteristic impedance of ${\underline{Z}}_{0}=50\;\Omega$. Reflection coefficient $S_{11}$ measurements are conducted at the entry point of the transmission line to determine the transformed input impedance by [51]

(6) $${\underline{Z}}_{\mathrm{RX},\mathrm{trns}}={\underline{Z}}_{\mathrm{NA}}\;\cdot \;\frac{1+S_{11}}{1-S_{11}}.$$

**Figure 11:**
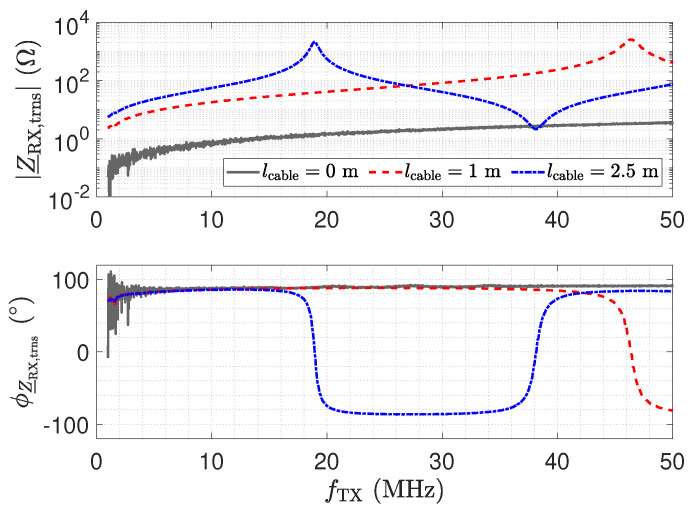
Measured magnitude $\left|{\underline{Z}}_{\mathrm{RX},\mathrm{trns}}\right|$ and phase $\phi_{{\underline{Z}}_{\mathrm{RX},\mathrm{trns}}}$ of the transformed input impedance according for the low-Z receiver depicted in Figure 10, for three different transmission line lengths. The result for a length of 0 m corresponds to ${\underline{Z}}_{\mathrm{RX}}$. At the point where the blue trend and the gray trend of $\left|{\underline{Z}}_{\mathrm{RX},\mathrm{trns}}\right|$ meet, a λ/2 setup is obtained.

Here, ${\underline{Z}}_{\mathrm{NA}}=50\;\Omega$ denotes the reference impedance of the used network analyser. Figure 11 shows the measurement result for ${\underline{Z}}_{\mathrm{RX},\mathrm{trns}}$. For a direct connection (0 m) in Figure 11, the amplifier maintains a low impedance with an inductive behaviour, which is caused by the operational amplifier used and the short copper trace on the printed circuit board (PCB) [26]. This behaviour for ${\underline{Z}}_{\mathrm{RX}}$ is considered good for the realization of a low-Z receiver.

In the frequency response plot of the impedance for a 2.5 m long transmission line, two resonances can be observed at about 19 MHz and 38 MHz. The maximum impedance at 19 MHz is caused by a λ/4 transformer, which transforms the low input impedance ${\underline{Z}}_{\mathrm{RX}}$ of the receiver into a high impedance [52] by

(7) $${\underline{Z}}_{\mathrm{RX},\mathrm{trns}}=\frac{{\underline{Z}}_{0}^{2}}{{\underline{Z}}_{\mathrm{RX}}}.$$

As a result, the properties of the receiver are lost at the sensor. For the 2.5 m long transmission line, the second resonance at 38 MHz is due to a second λ/4 transformer effect. The resulting λ/2 transformer provides

(8) $${\underline{Z}}_{\mathrm{RX},\mathrm{trns}}={\underline{Z}}_{\mathrm{RX}}.$$

Hence, for a line length of λ/2, the original input impedance of the low-Z receiver appears at the sensor. This can also be observed by the blue impedance trend in Figure 10, which matches the original trend without a line. Thus, for the λ/2 transformer, the transmission line has no influence. In the frequency response plot of the impedance for a 1 m long transmission line, only one resonance can be observed, which is for the corresponding λ/4 transformer. The λ/2 transformer is not obtained in the measurement.

While the λ/4 and λ/2 transformer form dedicated points in the operation of the receiver with the cable, it is remarkable that, also for low frequencies, e.g., for 1 MHz, a significant variation in the impedance appears. ${\underline{Z}}_{\mathrm{RX},\mathrm{trns}}$ is still low with respect to the impedance of $C_{s1}$; e.g., at 1 MHz, the impedance is in the range of 16  KΩ. However, the measurement shows the considerable influence of the line.

All resonance frequencies lengths are in good agreement with the theoretical values, which can be determined by

(9) $$\lambda =\frac{c}{VF\;\cdot \;f}$$

Here, *c* is the speed of light in vacuum, and VF is the velocity factor, which is 0.66 for the RG174 [53] cable used. Generally, the impedance transformation due to a transmission line can be computed by [52]

(10) $${\underline{Z}}_{\mathrm{RX},\mathrm{trns}}={\underline{Z}}_{0}\;\cdot \;\frac{{\underline{Z}}_{\mathrm{RX}}+i\;\cdot \;{\underline{Z}}_{0}\;\cdot \;\tan\left(\beta \;\cdot \;l_{\mathrm{cable}}\right)}{{\underline{Z}}_{0}+i\;\cdot \;{\underline{Z}}_{\mathrm{RX}}\;\cdot \;\tan\left(\beta \;\cdot \;l_{\mathrm{cable}}\right)}.$$

**Figure 12:**
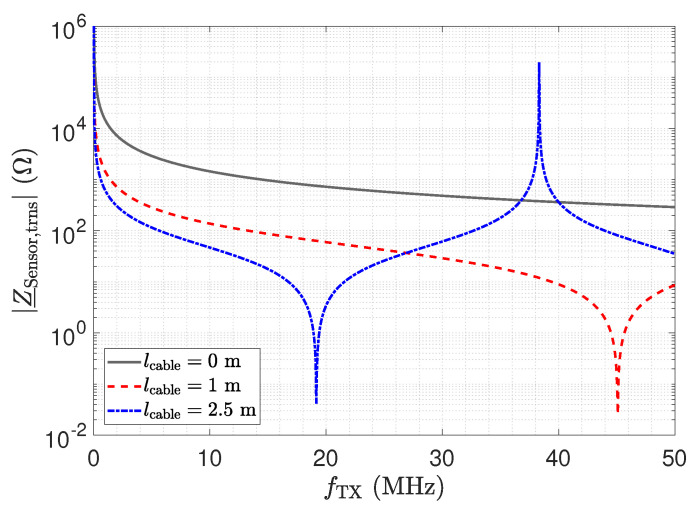
Analytical result of the transformed sensor impedance ${\underline{Z}}_{\mathrm{Sensor},\mathrm{trns}}$, as depicted in Figure 10. The result for a length of 0 m corresponds to ${\underline{Z}}_{\mathrm{Sensor}}$. Close to 40 MHz, where the blue trend and the red trend meet, a λ/2 setup is obtained.

$\beta (f_{\mathrm{TX}})$ represents the frequency dependent phase constant of the transmission line. It can be determined from

(11) $$\gamma =\alpha +i\beta =\sqrt{\left(R^{{}^{\prime }}+i2\pi f_{\mathrm{TX}}L^{{}^{\prime }}\right)\;\cdot \;\left(G^{{}^{\prime }}+i2\pi f_{\mathrm{TX}}C^{{}^{\prime }}\right)}.$$

where α is the attenuation constant and γ is the propagation constant. The resistance, inductance, conductance, and capacitance per unit length are represented by $R^{\prime }$, $L^{\prime }$, $G^{\prime }$, and $C^{\prime }$, respectively. Equation (10) can also be used to analyse the impedance ${\underline{Z}}_{\mathrm{Sensor},\mathrm{trns}}$, which is shown in Figure 12. For the line, the parameters $G^{\prime }=0\frac{S}{m}$, $R^{\prime }=1\frac{\Omega }{m}$, $L^{\prime }=250\frac{\mathrm{nH}}{m}$, and $C^{\prime }=100\frac{pF}{m}$ have been used. At λ/2, the impedance ${\underline{Z}}_{\mathrm{Sensor},\mathrm{trns}}$ equals ${\underline{Z}}_{\mathrm{Sensor}}$. Note that the frequency for the λ/2 is slightly shifted with respect to the measurement. We attribute this to the deviation of the RG174 cable from the ideal behaviour, which was used in the simulation.

### 3.2. Low-Z Receiver: Output Behaviour

**Figure 13:**
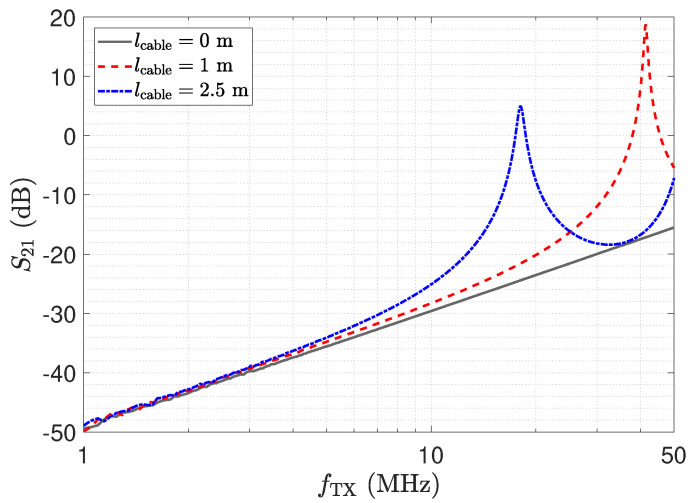
Measurement results for three different cable lengths for the circuit in Figure 10, determined by a network analyser.

In this section, we address the output behaviour of the low-Z receiver. For the investigation, we performed measurements of the transmission coefficient $S_{21}$ of the circuit depicted in Figure 10. Therefore, port 1 of the network analyser is attached to the left side of the lumped Π-network shown in Figure 10 and port 2 measures $V_{\mathrm{RX}}$. Measurement results for three different transmission line lengths are shown in Figure 13. The power of the excitation was set to −20 dBm at 50 Ω. The frequency response determined for a cable length of 0 m represents the direct attached assembly investigated in Section 2.

For the direct attached assembly the stray capacitance $C_{s1}$ has no effect on the determined frequency response of the transmission coefficient, thus providing a linear behaviour. For the measurement with the transmission lines, again distinct peaks can be observed at the frequencies corresponding to the λ/4 transformer for the specific lines. It is important to understand that, although a larger measurement signal is provided, measurements should not be taken at these frequencies. The impedance ${\underline{Z}}_{\mathrm{RX},\mathrm{trns}}$ at the sensor is not low but transformed to a high value as described by Equation (7). Hence, $C_{s1}$ is not shunted. This deviates from the low-Z measurement scheme and leads to higher cross sensitivities in the sensor with respect to ${\underline{Z}}_{\mathrm{RX},\mathrm{trns}}$. Yet for $l_{\mathrm{cable}}=2.5\;m$ at 39 MHz, the same behaviour as for the direct connection can be observed due to the λ/2 transformer. Thus, only for the λ/2 setup is the signal path well defined, and this should be used for measurements with the low-Z receiver with transmission lines.

To show the behaviour of the operational amplifier, we studied the signals $V_{\mathrm{TL},\mathrm{out}}$ and $I_{\mathrm{TL},\mathrm{out}}$ as defined in Figure 10. From the measurements, we evaluate $I_{\mathrm{TL},\mathrm{out}}$ by

(12) $$I_{\mathrm{TL},\mathrm{out}}=-\frac{V_{\mathrm{RX}}}{R_{f}}=-\frac{\frac{V_{\mathrm{TX}}\;\cdot \;S_{21}}{2}}{R_{f}}.$$

and ${\underline{V}}_{\mathrm{TL},\mathrm{out}}$ by

(13) $${\underline{V}}_{\mathrm{TL},\mathrm{out}}=I_{\mathrm{TL},\mathrm{out}}\;\cdot \;{\underline{Z}}_{\mathrm{RX}}.$$

The input impedance ${\underline{Z}}_{\mathrm{RX}}$ of the transimpedance amplifier is taken from the measurements. $R_{f}$ denotes the feedback resistor. For a second approach, which we refer to as the analytic approach, we evaluate the quantities using the relations

(14) $$\underline{V}\left(z,z^{\prime }\right)={\underline{V}}_{\mathrm{ini}}\;e^{-\gamma z}\left(\frac{1+\Gamma_{\mathrm{RX}}\;e^{-2\gamma z^{\prime }}}{1-\Gamma_{\mathrm{Sensor}}\;\Gamma_{\mathrm{RX}}\;e^{-2\gamma l_{\mathrm{cable}}}}\right),$$

(15) $$\underline{I}\left(z,z^{\prime }\right)=\frac{{\underline{V}}_{\mathrm{ini}}}{{\underline{Z}}_{0}}\;e^{-\gamma z}\;\left(\frac{1-\Gamma_{\mathrm{RX}}\;e^{-2\gamma z^{\prime }}}{1-\Gamma_{\mathrm{Sensor}}\;\Gamma_{\mathrm{RX}}\;e^{-2\gamma l_{\mathrm{cable}}}}\right),$$

which describe the transformation of the voltage and the current along the transmission line. ${\underline{V}}_{\mathrm{ini}}$ is the input voltage of the line [54]. $\Gamma_{\mathrm{RX}}$ and $\Gamma_{\mathrm{Sensor}}$ are the reflection coefficients of the sensor and the transimpedance amplifier, respectively. They are computed by

(16) $$\Gamma_{i}=\frac{{\underline{Z}}_{i}-{\underline{Z}}_{0}}{{\underline{Z}}_{i}+{\underline{Z}}_{0}}.$$

**Figure 14:**
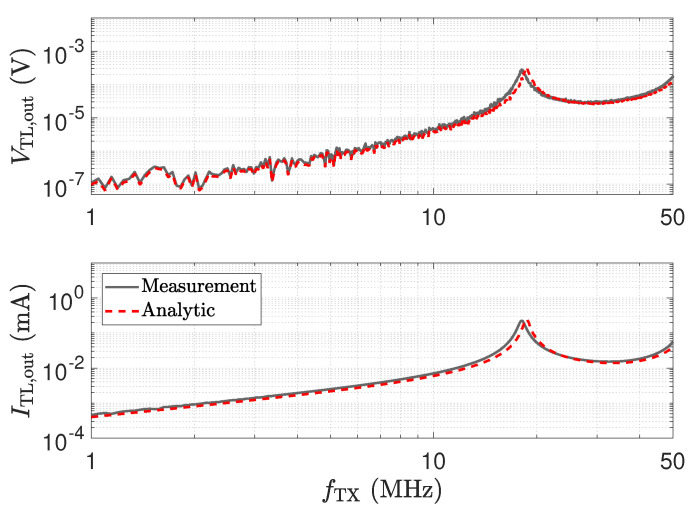
Voltage and current peaking at the output of a 2.5 m long transmission line, as depicted in Figure 10. The measurement result of a direct attached assembly ($l_{\mathrm{cable}}=0\;m$), shown in Figure 11, is used to determine the trends denoted by “Analytic”, according to Equations (14)–(16), with $G^{\prime }=0\frac{S}{m}$, $R^{\prime }=1\frac{\Omega }{m}$, $L^{\prime }=250\frac{\mathrm{nH}}{m}$ and $C^{\prime }=100\frac{\mathrm{pF}}{m}$. The frequency responses marked by “Measurement” are determined by the use of the transfer coefficient $S_{21}$ in Figure 13.

$\Gamma_{\mathrm{RX}}$ is evaluated from the measurement of the ${\underline{Z}}_{\mathrm{RX}}$ of the transimpedance-amplifier. For the sensor, we used the equivalent circuit model as mentioned above. The results for the cable length of 2.5 m are depicted in Figure 14 and show a good agreement for the two approaches. The result coincides with the transmission measurement depicted in Figure 13. For the λ/4 transformer, the current shows a peak, which leads to the high output value. However, the high value of ${\underline{V}}_{\mathrm{TL},\mathrm{out}}$ of the opamp indicates an unfavourable operating condition. Thus, in addition to the higher cross-sensitivity in the sensor, there is also a higher strain on the component, showing again that the peaking for λ/4 should not be used for measurement. For the low-Z receiver, a λ/2 setup has to be used.

### 3.3. Low-Z Receiver: Noise Characteristics

**Figure 15:**
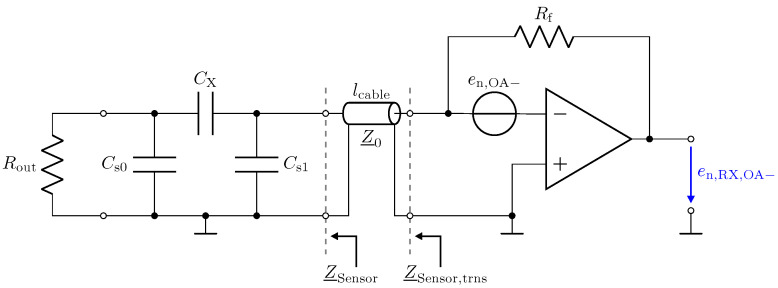
Circuit configuration of the low-Z input stage to determine its output voltage noise. As an example, the voltage noise spectral density $e_{n,\mathrm{OA}-}$ of the inverting amplifier input and its resulting output voltage noise spectral density $e_{n,\mathrm{RX},\mathrm{OA}-}$ are shown. $R_{\mathrm{out}}=50\;\Omega$, $R_{f}=500\;\Omega$, $C_{X}=1\;pF$ and $C_{s0}=C_{s1}=10\;pF$.

In this section, we present a noise analysis for the low-Z input stage in combination with transmission lines. Therefore, a SPICE simulation is performed, including the amplifier model provided by the manufacturer. Due to the inverting amplifier structure, the current noise at the positive input of the opamp causes no contribution to the noise voltage at the output. The current at the negative input flows over the feedback resistance $R_{f}$, where it contributes with a constant noise floor. However, with respect to its effect on the output, the noise voltage of the opamp must be examined more closely. Figure 15 shows the investigated configuration for this noise analysis. The source $e_{n,\mathrm{OA}-}$ at the negative input presents the spectral voltage noise density. For its analysis, the capacitive Π-network and the transmission line must be considered as well.

**Figure 16:**
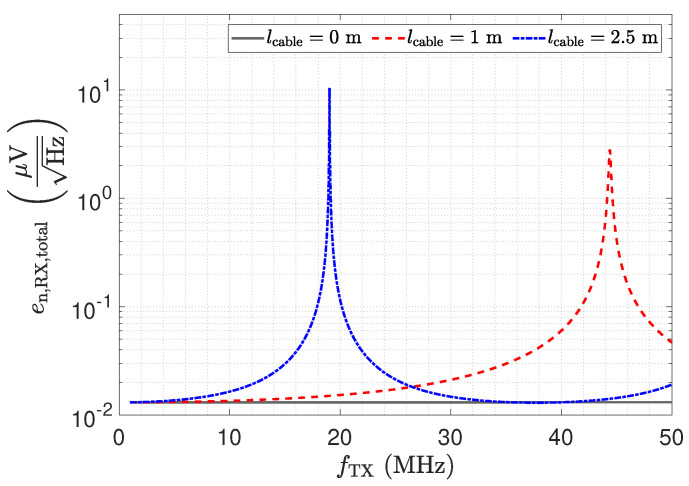
Total output voltage noise spectral density of the investigated low-Z circuit for three different transmission line lengths. The SPICE based simulation includes the capacitive Π network.

Figure 16 shows the resulting total voltage noise spectral density $e_{n,\mathrm{RX},\mathrm{total}}$ for three transmission line configurations. For the presented setup and opamp, we found that the voltage noise is the dominant noise source. Therefore, although the results depicted in Figure 16 show the total noise, they are representative of the discussion of the voltage noise. The two peaks in Figure 16 for a 1 m and a 2.5 m long cable are again a result of the impedance transformation property of the transmission line. The spectral noise density $e_{n,\mathrm{OA}-}$ is amplified by

(17) $$e_{n,\mathrm{RX},\mathrm{OA}-}=e_{n,\mathrm{OA}-}\;\cdot \;\left|1+\frac{R_{f}}{{\underline{Z}}_{\mathrm{Sensor},\mathrm{trns}}}\right|.$$

For λ/4 the impedance ${\underline{Z}}_{\mathrm{Sensor},\mathrm{trns}}$ becomes small, as shown in Figure 12, leading to the significant amplification of the noise. For λ/2, the noise floor reaches the same low value as for a direct connection.

## 4. Matched Receiver

**Figure 17:**
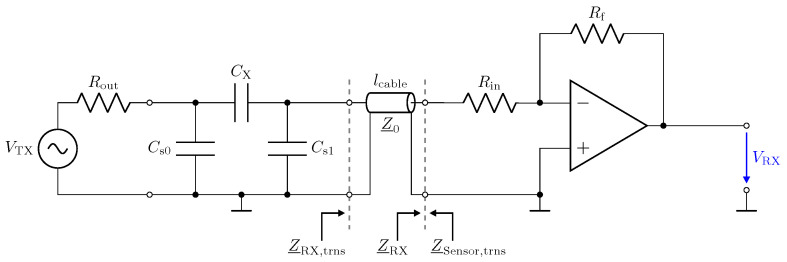
Matched receiver circuit connected to a capacitive Π-network by means of a transmission line with length $l_{\mathrm{cable}}$ and impedance ${\underline{Z}}_{0}=50\;\Omega$. $R_{\mathrm{in}}=R_{\mathrm{out}}=50\;\Omega$, $R_{f}=500\;\Omega$, $C_{X}=1\;pF$, $C_{s0}=C_{s1}=10\;pF$.

In this section, we address the matched receiver. Figure 17 shows a circuit realization of the matched input stage illustrated in Figure 4b. The inverting operational amplifier includes an input resistance $R_{\mathrm{in}}$, which meets the characteristic wave impedance ${\underline{Z}}_{0}$ of 50 Ω. By providing a defined input resistance of 50 Ω, high-frequency effects such as impedance transformation and standing waves are avoided.

### 4.1. Matched Receiver: Behaviour of ${\underline{Z}}_{\mathrm{RX},\mathrm{trns}}$ and Output Behaviour

**Figure 18:**
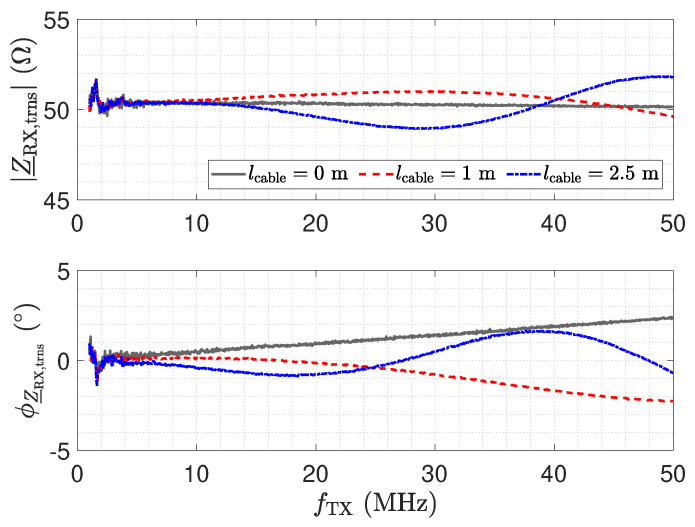
Measured magnitude $\left|{\underline{Z}}_{\mathrm{RX},\mathrm{trns}}\right|$ and phase $\phi_{{\underline{Z}}_{\mathrm{RX},\mathrm{trns}}}$ of the transformed input impedance according to Figure 17 for three different transmission line lengths. The result for a length of 0 m corresponds to ${\underline{Z}}_{\mathrm{RX}}$.

Figure 18 shows the measurement results of the transformed receiver input impedance for the three different cable lengths, determined by a network analyser. The measurement result for a cable length $l_{\mathrm{cable}}$ of 0 m represents the non-transformed input impedance ${\underline{Z}}_{\mathrm{RX}}$, as depicted in Figure 17. The matched receiver structure maintains an input impedance close to the desired 50 Ω for the investigated frequency range. The deviations from 50 Ω are actually due to the tolerances of the ${\underline{Z}}_{0}$ of the transmission line [53].

**Figure 19:**
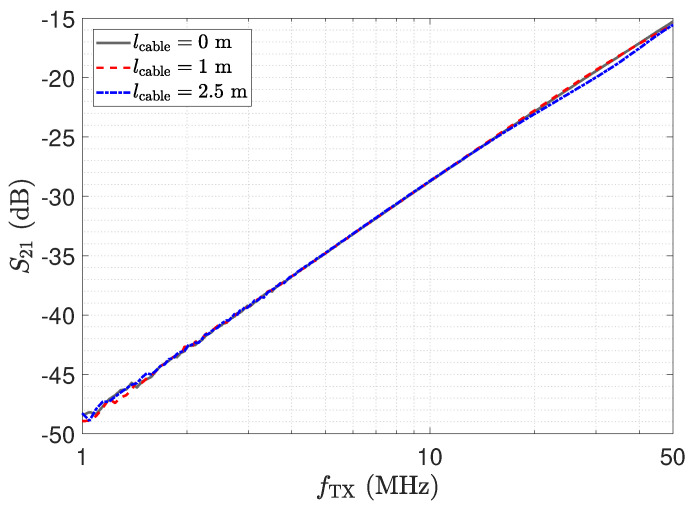
Measurement results for three different cable lengths for the circuit in Figure 17, determined by a network analyser.

Transfer coefficient $S_{21}$ measurement results for the matched input stage are shown in Figure 19. The result for a cable length of 0 m, corresponds to the analytical result presented for direct attached assembly in Figure 5, investigated in Section 2. As can be seen, the transfer performance of the matched circuit is almost independent of the transmission line length used. The minor deviations can be compensated by calibration.

### 4.2. Matched Receiver: Noise Characteristics

**Figure 20:**
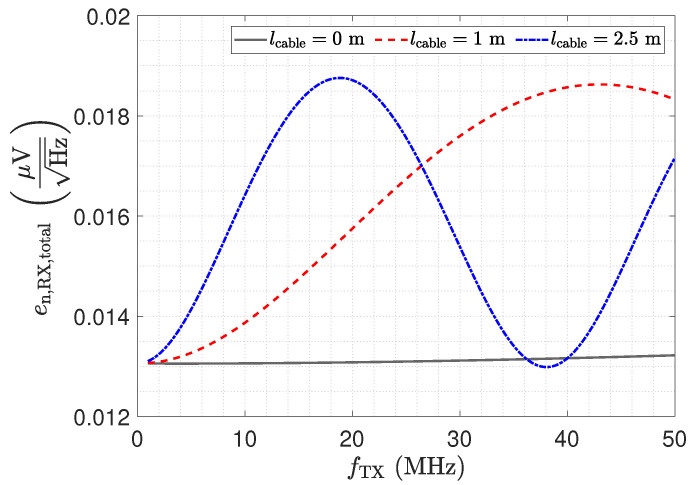
Total output voltage noise spectral density of the investigated matched input stage for three different transmission line lengths. The SPICE-based simulation includes the capacitive Π-network.

The SPICE-based noise simulation results of the matched input stage including the capacitive Π-network are illustrated in Figure 20. For the direct connection, a constant noise floor is obtained. The noise floor equals the noise floor of the low-Z receiver for a direct connection. The oscillations for the noise floor when using cables can again be explained by the impedance ${\underline{Z}}_{\mathrm{Sensor},\mathrm{trns}}$, which is depicted in Figure 12. Yet the resistance $R_{\mathrm{in}}$ limits the amplification of the noise. This can also be seen by Equation (17), where the resistance $R_{\mathrm{in}}$ appears in the denominator, limiting the amplification to the gain of the amplifier.

## 5. LCR Receiver

The LCR input stage has been suggested for a direct attached assembly of the sensor and the electronics [36]. In contrast to the other two circuits, this circuit is tuned to a specific measuring frequency. Therefore, the discussion in this section has a different structure.

### 5.1. Design of a Matched LCR Receiver Input Stage

**Figure 21:**
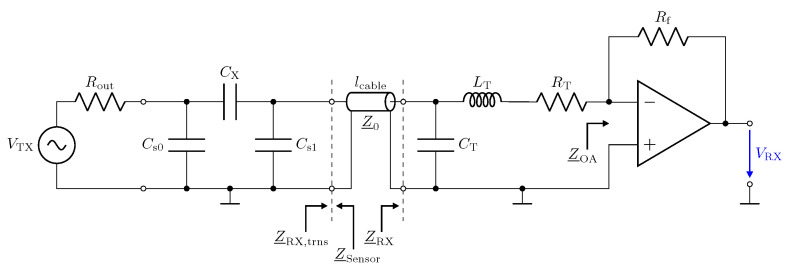
Resonant receiver circuit connected to a capacitive Π-network by means of a transmission line with length $l_{\mathrm{cable}}$ and impedance ${\underline{Z}}_{0}=50\;\Omega$. $R_{\mathrm{out}}=50\;\Omega$, $R_{f}=500\;\Omega$, $C_{X}=1\;pF$, $C_{s0}=C_{s1}=10\;pF$.

In this section, we address the design of an LCR receiver input stage, which has an input impedance of ${\underline{Z}}_{0}$ at its resonance frequency. We refer to this as matched LCR receiver. The circuit implementation of the resonant LCR input stage, illustrated in Figure 4c, is shown in Figure 21. The capacitance $C_{T}$ and the inductance $L_{T}$ form a parallel circuit. At the resonance frequency

(18) $$f_{0}=\frac{1}{2\;\cdot \;\pi \;\cdot \;\sqrt{L_{T}\;\cdot \;C_{T}}}$$

the input impedance of an ideal parallel resonant circuit is high. This is actually not suited for the capacitive measurement, yet because of the resistance $R_{T}$, the real resonance circuit can have low impedance values. Note that the resistance $R_{T}$ also leads to a shift of the resonance frequency by [55,56]

(19) $$f_{p}=f_{0}\;\cdot \;\sqrt{1-\frac{1}{Q^{2}}}.$$

where the quality factor *Q* (Q factor) is given by

(20) $$Q=\frac{1}{R_{T}}\;\cdot \;\sqrt{\frac{L_{T}}{C_{T}}}.$$

For the application of the LCR input stage with a transmission line, we design the input stage to have an input impedance of ${\underline{Z}}_{0}=50\;\Omega$. Following [36], we design the circuit for a resonance frequency of 40 MHz. $L_{T}$ and $C_{T}$ can then be determined by

(21) $$L_{T}=\sqrt{\frac{{\underline{Z}}_{0}\;\cdot \;R_{T}-R_{T}^{2}}{{\left(2\;\cdot \;\pi \;\cdot \;f_{\mathrm{TX}}\right)}^{2}}}$$

and

(22) $$C_{T}=\frac{L_{T}}{{\underline{Z}}_{0}\;\cdot \;R_{T}}.$$

With a resistance of 1 Ω for $R_{T}$, we obtain a capacitance of about 557 pF and an inductance of 27.85 nH. The *Q* factor is $Q_{\mathrm{matched}}=7.36$.

**Figure 22:**
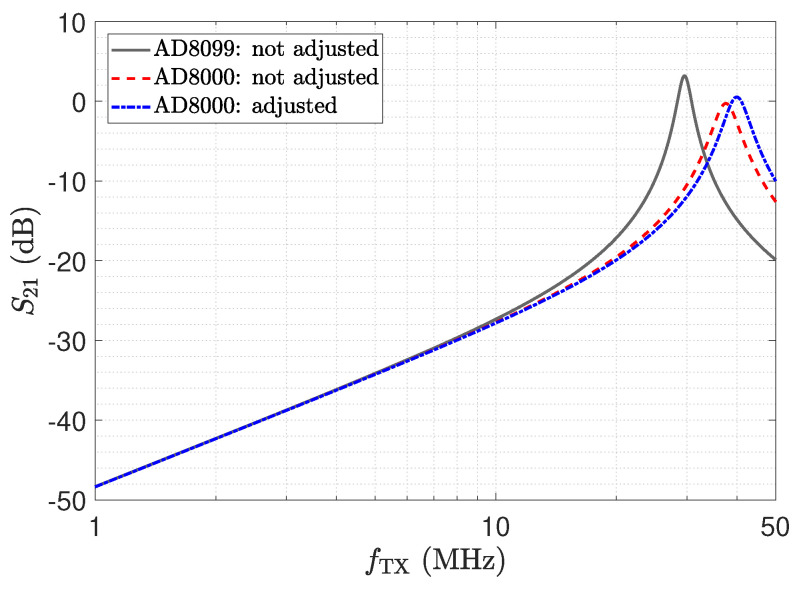
Transfer function of the matched LCR circuit determined by a SPICE simulation obtained for a direct attached assembly, as discussed in Section 2. The simulations were performed for two different operational amplifiers.

The resulting frequency responses of the transfer coefficient for a direct attached assembly ($l_{\mathrm{cable}}$= 0 m), determined by a SPICE simulation, are shown in Figure 22. Note that, for a directly attached assembly, the parasitic stray capacitance $C_{s1}$, as depicted in Figure 3, has to be considered, which leads to a lumped capacitance of ${\tilde{C}}_{T}=C_{T}-C_{s1}$. The simulations have been performed for two different operational amplifiers. The AD8099 from Analog Devices [57] has been suggested for this kind of receiver topology by [36,58]. The good input impedance performance of the AD8000 is shown in Section 3 and Section 4.

**Figure 23:**
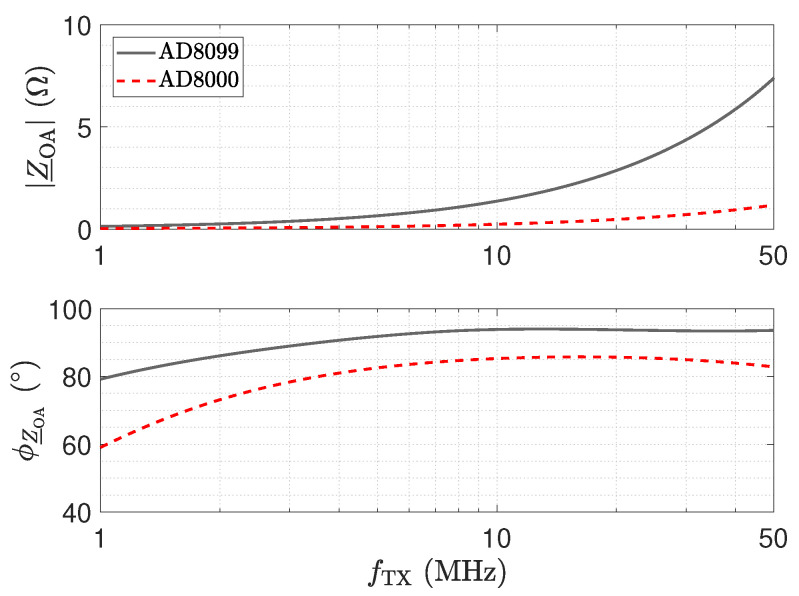
Input impedance magnitude $\left|{\underline{Z}}_{\mathrm{OA}}\right|$ and phase $\phi_{{\underline{Z}}_{\mathrm{OA}}}$ for two different operational amplifiers, as depicted in Figure 21. The impedances have been determined by a SPICE simulation.

For the grey and red trends depicted in Figure 22, which are labelled “not adjusted”, a significant deviation from the desired resonant frequency of 40 MHz can be recognized. We identified the cause for this shift by the input impedance ${\underline{Z}}_{\mathrm{OA}}$ of the amplifier, which we marked in Figure 21. Figure 23 shows an analysis of ${\underline{Z}}_{\mathrm{OA}}$ for the two amplifiers. ${\underline{Z}}_{\mathrm{OA}}$ is of inductive character; e.g., for the AD8000, an equivalent input inductance and resistance of 3.72 nH and 109 mΩ can be determined for 40 MHz.

By adjusting $L_{T}$, the blue trend in Figure 22 can be obtained, where the circuit shows the desired behaviour with respect to the resonance frequency. Note that to meet the intended resonance frequency, printed circuit board (PCB) parasitics [59,60], as well as the parasitics of the used lumped components, have to be considered [61,62]. We also studied the temperature behaviour of this circuit in SPICE simulations and found that ${\underline{Z}}_{\mathrm{OA}}$ has a significant temperature dependency. The SPICE simulations have been carried out at 27 °C. For 85 °C, we obtained a frequency shift of 0.3 MHz towards lower frequencies. The shift results in a reduction in the transfer coefficient $S_{21}$ of approximately 0.3 dB. With respect to measurement applications in harsh environments, this aspect should not be overlooked. In contrast, the other circuits show a robust behaviour with respect to this property.

### 5.2. LCR Receiver: Behaviour of ${\underline{Z}}_{\mathrm{RX},\mathrm{trns}}$

**Figure 24:**
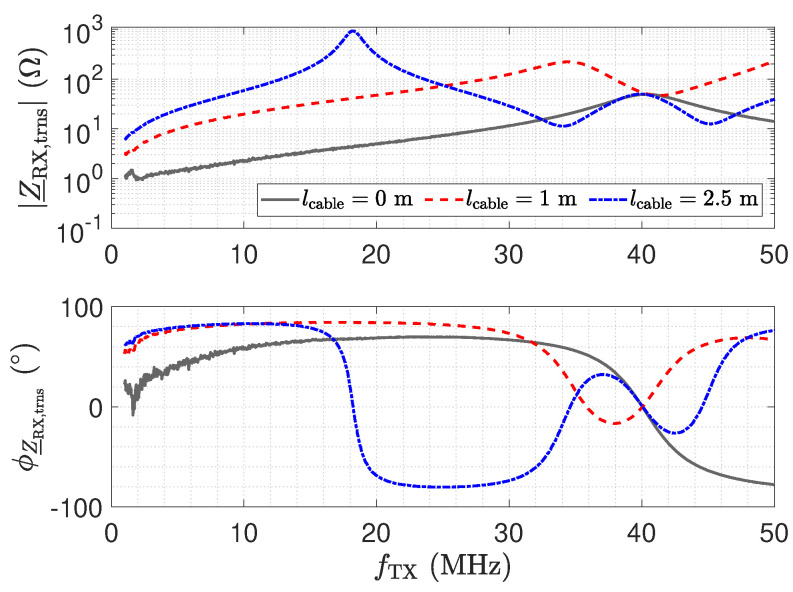
Measured magnitude $\left|{\underline{Z}}_{\mathrm{RX},\mathrm{trns}}\right|$ and phase $\phi_{{\underline{Z}}_{\mathrm{RX},\mathrm{trns}}}$ of the transformed matched LCR input impedance according to Figure 21, for three different transmission line lengths. The result for a length of 0 m corresponds to ${\underline{Z}}_{\mathrm{RX}}$. The impedance magnitude at 40 MHz is about 50 Ω for all three cable lengths. The corresponding phase at 40 MHz is close to 0 °.

**Figure 25:**
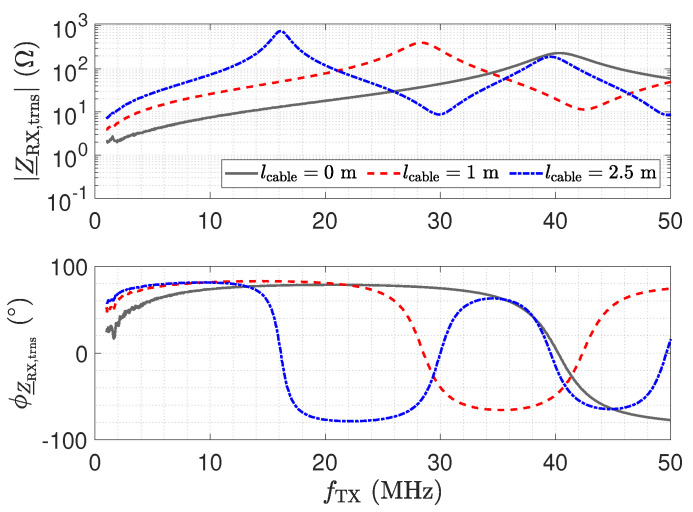
Measured magnitude $\left|{\underline{Z}}_{\mathrm{RX},\mathrm{trns}}\right|$ and phase $\phi_{{\underline{Z}}_{\mathrm{RX},\mathrm{trns}}}$ of the transformed input impedance according to Figure 21, for three different transmission line lengths. The result for a length of 0 m corresponds to ${\underline{Z}}_{\mathrm{RX}}$.

In this section, we present measurements of ${\underline{Z}}_{\mathrm{RX},\mathrm{trns}}$ for the matched LCR receiver. In addition, we present measurements for an LCR receiver, which is not matched. The component values have been taken from [36], where the receiver designed for a direct connection. Figure 24 and Figure 25 present the measurements of ${\underline{Z}}_{\mathrm{RX},\mathrm{trns}}$ for the matched and the non-matched design, respectively. For the matched design, we observe a good agreement between ${\underline{Z}}_{\mathrm{RX},\mathrm{trns}}$ for a direct connection and for both cable lengths at the resonance frequency. In contrast to the low-Z receiver, the cable length is a degree of freedom for the matched LCR receiver.

For the measurements depicted in Figure 25, the unmatched receiver has an input impedance of about 110 Ω. The *Q* factor of this receiver input network is Q=16.36. The impact of this mismatch can be directly observed. For the line length of 1 m, a significant impedance transformation is present. For the line length of 2.5 m, a deviation from the behaviour of the matched receiver can be observed.

### 5.3. LCR Receiver: Output Behaviour

**Figure 26:**
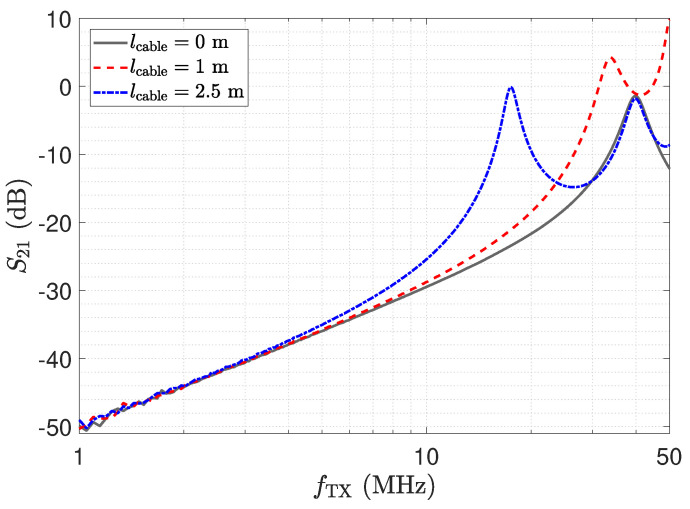
Measurement results for three different cable lengths of the matched version of the resonant LCR circuit shown in Figure 21. As the circuit input impedance in Figure 24 is close to 50 Ω at 40 MHz, almost the same transfer coefficient is obtained at 40 MHz for all three cable lengths.

In this section, measurement results for the transfer coefficient $S_{21}$ of the matched and the not-matched LCR receiver are presented. Figure 26 depicts the measurement result for the matched receiver. At the resonance frequency, again, all trends meet.

**Figure 27:**
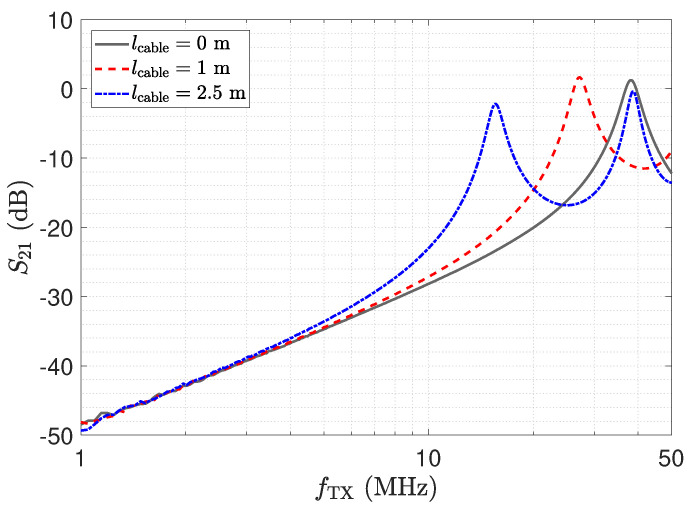
Measurement results for three different cable lengths for the circuit in Figure 21, determined by a network analyser. The input stage of the LCR receiver was not matched to the transmission line. For the λ/2 setup, the blue and the gray trends meet.

A second peak with equal signal strength can be observed at the λ/4 transformer for the 2.5 m line. However, this point is again not suitable for measurements. At this frequency, the input impedance of the LCR receiver deviates from ${\underline{Z}}_{0}$, so the transmission line is not correctly terminated at the output. The impedance ${\underline{Z}}_{\mathrm{RX},\mathrm{trns}}$ at the sensor is high. This again increases the cross-sensitivity with respect to $C_{s1}$ as this capacitance is not shunted. Figure 27 shows the transfer coefficient measurements for the LCR receiver when it is not matched. While the behaviour is similar, a deviation from the direct connection can be observed for the 2.5 m line at the resonance frequency as a result of the not-well-defined signal propagation. For the line length of 1 m, we observe a significant deviation from the desired behaviour; e.g., the resonance peak appears below 30 MHz. The line causes a significant detuning effect.

**Figure 28:**
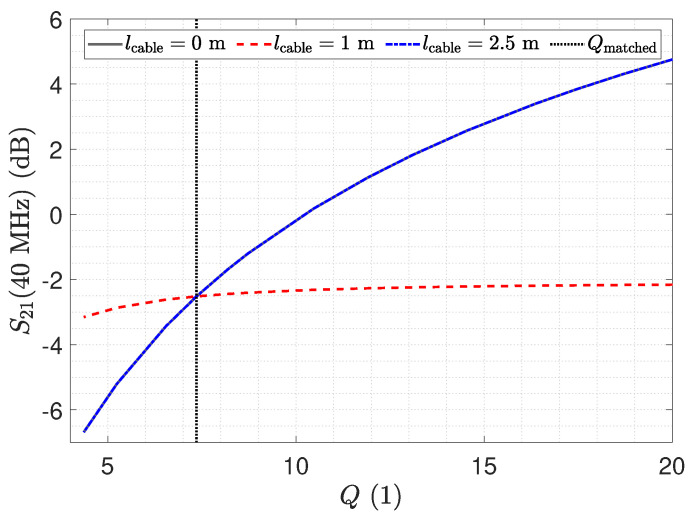
Transmission coefficient $S_{21}$ at 40 MHz as a function of the LCR circuit quality factor determined by a SPICE simulation. $Q_{\mathrm{matched}}$ denotes the quality factor of the matched LCR circuit.

Figure 28 shows the transfer coefficient $S_{21}$ at 40 MHz as a function of the quality parameter *Q* of the LCR receiver input stage. The diagram is the result of a SPICE simulation. For the line length of 2.5 m, the transfer coefficient $S_{21}$ can be increased by the quality factor *Q*, as this line length is close to the λ/2 transformer for a set resonance frequency. However, an increased value of *Q* leads to higher input impedance of the receiver stage and hence to a reduced shunting of $C_{s1}$. For the line length of 1 m, the setup is close to that of a λ/4 transformer. Here, a change in the quality factor *Q* shows no distinct improvement, which we understand to be caused again by the detuning due to the line.

### 5.4. Resonant LCR Receiver: Noise Characteristics

**Figure 29:**
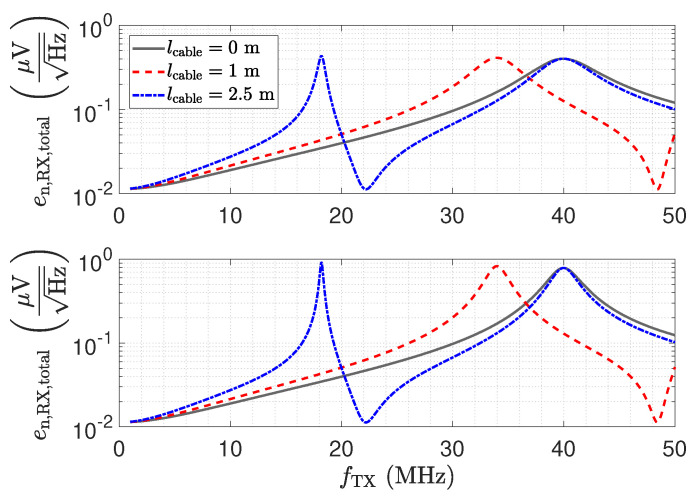
Upper plot: Total output voltage noise spectral density of the investigated matched resonant LCR input stage with $Q_{\mathrm{matched}}=7.36$, for three different transmission line lengths. Lower plot: Total output voltage noise spectral density of a non-matched resonant LCR input stage with Q=16.36, for three different transmission line lengths. The SPICE-based simulation includes the capacitive Π network.

As for the other receiver structures, we performed SPICE-based noise simulations for the LCR receiver. Figure 29 depicts the noise density at the output for the matched and the unmatched LCR receiver. The noise floor for the line length of 2.5 m and the noise floor for the directly connected receiver meet at the resonance frequency. This setup is close to the λ/2 setup. While the resonance circuitry of the LCR receiver elevates the signal as depicted in Figure 26, it also leads to an increased noise floor. This arises due to the series resonance acting for the noise voltage. With a line length of 1 m, there is a frequency shift of the maximum value. This can be explained by the impedance ${\underline{Z}}_{\mathrm{RX},\mathrm{trns}}$, which is depicted in Figure 12. This impedance causes a detuning of the resonance circuit, which leads to the different behaviour.

## 6. Receiver Structure Assessment

In the Section 3, Section 4 and Section 5, the three different receiver types in combination with transmission lines were analysed. Their properties and different aspects have been addressed. In this section, we summarize the main aspects for a comparison. Finally, we address the receivers by means of a comparison of the SNR.

### 6.1. Receiver Operation Comparison 

#### 6.1.1. Low-Z Receiver

- Simple realization feasibility of the circuits possible with moderate effort.
- Even small transmission line lengths show a transformation of ${\underline{Z}}_{\mathrm{RX},\mathrm{trns}}$ to higher input impedances. This also increases the cross-sensitivity with respect to parasitic capacitances.
- Selection of frequency and transmission line length: the frequency and the transmission line length have to be matched to obtain a λ/2 transformer. In this case, the circuit behaves as for a direct connection except for the attenuation of the line. The attenuation can be calibrated.
- A λ/4 setup has to be avoided. Although it leads to high output signals, it creates undefined signal propagation and harmful operating conditions for the opamp
- Noise: Same noise floor as with direct connection for λ/2 transformer. Significant elevation of noise floor towards the λ/4 setup.

The low-Z receiver provides the best performance for a directly attached assembly. With transmission lines, only a λ/2 setup should be used. Hence, the selection of the measurement frequency and the line length is strongly related to each other. For this setup, it achieves the same properties as for a directly connected receiver.

#### 6.1.2. Matched Receiver

- Simple realization feasibility of the circuits possible with moderate effort. Variations of ${\underline{Z}}_{0}$ in the transmission line remain a minor source of deviations in the circuit behaviour.
- Selection of frequency and transmission line length. Due to the matched setup, the frequency can be selected independently from the transmission line length. The receiver provides a constant input impedance.
- Noise: the receiver structure shows an almost constant noise floor over the whole frequency range.

The input impedance of this receiver leads to a minor reduction in the signal, which was addressed in Section 2.1. However, this small deviation can be calibrated. In comparison to the low-Z receiver, the matched receiver provides a well-defined signal path, setting no constraints on the choice of the measurement frequency and the line length. This enables the matched receiver to be used in adopted measurement schemes, e.g., the application of frequency spectroscopic measurement schemes.

#### 6.1.3. LCR-Receiver

- Realization requires tuning of the input stage. The tuning is sensitive towards parasitic effects of the assembly and the opamp. Furthermore, a relevant sensitivity with respect to temperature changes was observed.
- Selection of frequency and transmission line length: the excitation frequency is a design parameter. If the input stage is tuned to ${\underline{Z}}_{0}$, the line length can be arbitrary. Otherwise, again, a λ/2 transformer setup should be used.
- Noise: the noise floor is also shaped by the LCR input stage. In a direct connection setup or a λ/2 setup, the noise floor increases towards the selected resonance frequency. Different line lengths can lead to a spectral shift of the rise in the noise floor.

In contrast to the low-Z receiver, the LCR receiver provides a degree of freedom with respect to the length of the transmission line when it is matched. However, we found the tuning of the resonance circuitry to be quite sensitive. An automated tuning, e.g., by means of varactor diodes, as was suggested in [26,30], for directly attached sensors is only of limited benefit, as the input impedance of the circuit should also be matched to ${\underline{Z}}_{0}$.

#### 6.1.4. Summary of Relevant Signal-Propagation and Noise Effects

Table 1 provides a summary of the relevant observations and effects for the amplifier concepts. For each investigated case, the relevant measurement and simulation results are listed. Most of the observations are derived for the low-Z receiver, as the effects are most pronounced in this circuit. Furthermore, the matched receiver is included. The results for the LCR receiver are not included, as it requires the careful tuning of the input circuitry.

In the following, the relevant points are briefly discussed, and the coherence between the measurements and the simulation results is highlighted.

1. The impedance $\left|{\underline{Z}}_{\mathrm{RX},\mathrm{trns}}\right|$ depicted in Figure 11 equals the input impedance of the low-Z amplifier for a direct connection. Figure 13: The input–output behaviour of the circuit with the transmission line equals a direct connection. Figure 12: The impedance ${\underline{Z}}_{\mathrm{Sensor},\mathrm{trns}}$ with the transmission line equals the impedance for a direct connection.
2. Figure 11: $\left|{\underline{Z}}_{\mathrm{RX},\mathrm{trns}}\right|$ equals the input impedance for a direct connection. Thus, ${\underline{Z}}_{\mathrm{Sensor},\mathrm{trns}}$ equals ${\underline{Z}}_{\mathrm{Sensor}}$, leading to a minimum noise gain as stated by Equation (17). Figure 16: the noise floor equals the noise floor for a direct connection.
3. Figure 13: The peak in the transmission behaviour is due to an impedance transformation. This behaviour is not suitable for measurements.
4. Figure 11: $\left|{\underline{Z}}_{\mathrm{RX},\mathrm{trns}}\right|$ is high, which subsequently leads to a low impedance ${\underline{Z}}_{\mathrm{Sensor},\mathrm{trns}}$, as depicted in Figure 12. Thus, we observe a significant amplification of the noise as formulated by Equation (17).
5. Figure 18 shows a constant input impedance of the matched receiver. This leads to the output behaviour depicted in Figure 19.
6. The constant input impedance depicted in Figure 18 limits the amplification of the noise. In Equation (17), the resistor $R_{\mathrm{in}}$ is added to ${\underline{Z}}_{\mathrm{Sensor},\mathrm{trns}}$, which limits the noise amplification. This leads to the noise floor depicted in Figure 20.

### 6.2. SNR Comparison

In this section, we address the properties of the different receiver circuits by means of the SNR. This comparison naturally has to consider the different characteristics of the circuits; e.g., the low-Z receiver should only be used in a λ/2 setup, while the matched receiver allows measurements independently of frequency and cable length. Hence, for the low-Z receiver, the SNR should only be evaluated in one point, whereas the SNR of the matched receiver can be evaluated over a certain frequency range. Furthermore, for the LCR receiver, the SNR evaluation is only meaningful at the resonance frequency of its input stage.

Due to this aspect, a comparison based on the definition of the SNR

(23) $$\mathrm{SNR}=\frac{|V_{\mathrm{RX}}{|}^{2}}{|V_{n}{|}^{2}},$$

where $V_{\mathrm{RX}}$ is the output voltage and $V_{n}$ is the total noise voltage, v does not yield a fair assessment. In an actual measurement system, the receiver will be followed by a narrow band filter $H_{f}\left(if\right)$, which determines the system bandwidth. This filter can be realized as an analogue filter or as a digital filter. The latter is of interest for frequency spectroscopic systems, since the realization of a tunable analogue filter is challenging. Thus, we propose an SNR comparison based on

(24) $$\mathrm{SNR}=\frac{|V_{\mathrm{RX}}\left(if\right){|}^{2}}{\int_{f=0}^{\infty }{\left|H_{f}\left(if\right)e_{n,\mathrm{RX},\mathrm{total}}\left(if\right)\right|}^{2}df}\approx \frac{|V_{\mathrm{Rx}}\left(if\right){|}^{2}}{e_{n,\mathrm{RX},\mathrm{total}}{\left(if\right)}^{2}B_{f}}$$

$e_{n,\mathrm{RX},\mathrm{total}}$ is the total noise density at the output, as has been evaluated in the previous sections for the different receivers. The later approximation is valid due to the narrow band characteristic of the filter $H_{f}\left(if\right)$. $B_{f}$ denotes the effective noise bandwidth. Thus, by applying the logarithm, we can obtain

(25) $${\mathrm{SNR}}_{\mathrm{dB}}=\underset{{\mathrm{SNR}}_{\mathrm{rel}}}{\underset{︸}{{\left.10\log\left(\frac{|V_{\mathrm{RX}}\left(if\right){|}^{2}}{e_{n,\mathrm{RX},\mathrm{total}}{\left(if\right)}^{2}}\right)\right|}_{@P_{\mathrm{dBm},\mathrm{ref}}}}}+\left(P_{\mathrm{dBm}}-P_{\mathrm{dBm},\mathrm{ref}}\right)-10\log\left(B_{f}\right)$$

where we refer to the first expression as relative SNR, which we denote by ${\mathrm{SNR}}_{\mathrm{rel}}$. It can be directly obtained from a noise analysis and a simulation of the system, as discussed in the previous sections. The simulation is performed with an excitation power of $P_{\mathrm{dBm},\mathrm{ref}}$; e.g., in the previous simulations and experiments an excitation power of −20 dBm at 50 Ω (this corresponds to an RMS excitation of about 22.36mV). Therefore, the second term in Equation (25) expresses the gain in the SNR for a different excitation signal. Lastly, the third term in Equation (25) expresses the reduction in the SNR due to the bandwidth of the filter. Thus, the approach gives a fair comparison of the SNR properties of the different receivers by ${\mathrm{SNR}}_{\mathrm{rel}}$. The approach also gives access for estimating the SNR of a specific system, e.g., by setting the excitation signal strength $P_{\mathrm{dBm}}$ and the filter $H_{f}\left(if\right)$.

**Figure 30:**
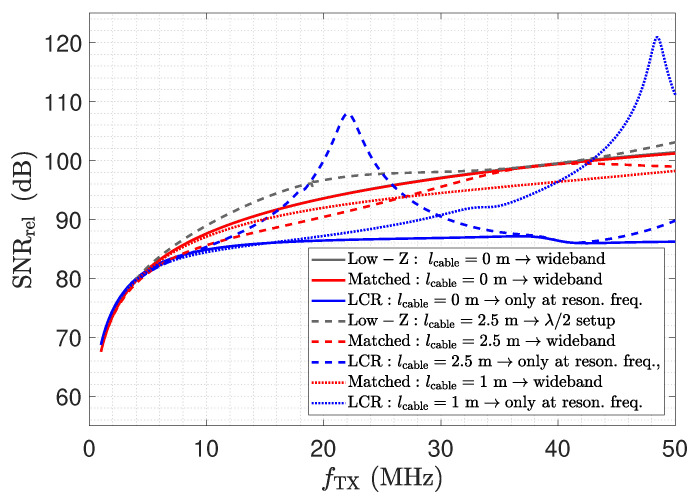
Comparison of ${\mathrm{SNR}}_{\mathrm{rel}}$ for the different receivers setups.

Figure 30 shows a comparison of the relative SNR of the three receivers for selected cable lengths. The legend also shows at which point the receiver should be used for correct operation. The bold lines depict ${\mathrm{SNR}}_{\mathrm{rel}}$ for the directly connected receivers. Here, no significant deviation between the low-Z and the matched receiver can be found, which is expected. The increasing relative SNR with respect to the frequency can be explained by the increased displacement current. For the LCR receiver, a reduced relative SNR can be observed. The evaluation has to be carried out at the resonance frequency of the input stage. Although the resonant input stage leads to an amplification of the output as shown in Figure 26, the noise floor also increases, as shown in Figure 29.

The results for a cable length of 2.5 m are depicted by the dashed lines. As expected, the low-Z receiver reaches the same relative SNR for the λ/2 setup. For frequencies below the λ/2 setup, the SNR is actually higher than for the directly attached setup. This behaviour follows the measurements of the transmission coefficient depicted in Figure 13, but as also the noise floor increases as depicted in Figure 16, the elevation of the relative SNR is moderate. Yet it has to be stated that an operation of the low-Z receiver at this point is not recommended. Furthermore, the relative SNR of the matched receiver shows a deviation from the directly attached setup. This behaviour coincides with the results of the noise analysis, e.g., the noise floor depicted in Figure 20.

For the matched LCR receiver, the relative SNR reaches the same level at the resonance frequency as for the directly attached assembly. This behaviour is expected, as the configuration is close to a λ/2 setup. Towards a frequency of 20 MHz, an elevation of the relative SNR can be observed. At this frequency, the LCR input stage does not provide an input impedance of ${\underline{Z}}_{0}$. Thus, the elevation of the SNR is again caused by undesired transmission line effects. This corresponds to the transmission measurement depicted in Figure 26.

Figure 30 further includes the results for the matched and the LCR receiver for a cable length of 1 m. For the matched receiver, a minor deviation is again visible, which can be attributed to the same cause as for the line length of 2.5 m. An interesting effect appears for the LCR receiver. While the output signal is the same, e.g., see Figure 26, due to the matched resonance circuit, the maximum value of the noise density is shifted towards a lower frequency, as depicted in Figure 29. This leads to an significant elevation of the SNR with respect to the direct connection and for the line length of 2.5 m, which is close to the λ/2 setup.

The results of the relative SNR analysis provide a suitable assessment of the different receivers and are in agreement with the previous results. The low-Z and the Matched receiver show a well-defined behaviour, and the selection of a circuit falls back to the points addressed in Section 6.1. Besides the addressed technical complexity of the LCR receiver, the lower SNR also shows a less favourable behaviour for this type of receiver. The elevation of the SNR by means of a transmission line element is an interesting option. We have not investigated this technique further, but we assume that this technique also requires careful adjustment of the circuitry. In particular, parasitic capacitances of the sensor also have to be considered.

## 7. Conclusions

In this paper, we have presented the analysis of different receiver topologies for capacitive measurement applications in combination with transmission lines. The analysis treats electrical and system aspects of the behaviour of the different circuit structures. The results definitely support the application of low-Z receiver structures or matched receiver structures. Low-Z receiver structures provide optimal behaviour in a λ/2 setup. However, this puts a constraint on the selection between the line length and the measurement frequency. In contrast, the matched receiver enables an independent choice for the selection of the frequency and the line length. The analysis of the LCR receiver has indicated several challenges and a reduced performance with respect to the SNR. The analysis approach and considerations provide researchers with a solid understanding of the receiver structures shown and indicate the necessary analysis steps for adapting further developments.

## Figures and Tables

**Table 1:** Summary of key observations and comparison between measurements and simulations.

Nr.	Description	Measurement	Simulation
1	low-Z Rec.: λ/2 effect	Figure 11, Figure 13	Figure 12
2	low-Z Rec.: λ/2 effect; noise	Figure 11	Figure 16, Figure 12, Equation (17)
3	low-Z Rec.: λ/4 effect	Figure 13	-
4	low-Z Rec.: λ/4 effect; noise	Figure 11	Figure 16, Figure 12, Equation (17)
5	Matched Rec.	Figure 19, Figure 18	-
6	Matched Rec.: noise	Figure 18	Figure 20, Equation (17)

## Abbreviations

The following abbreviations are used in this manuscript:

BPF	Band-pass filter
CF	Carrier frequency
ECT	Electrical capacitance tomography
GND	System ground
High-Z	High-impedance
IF	Intermediate frequency
LCR	Inductor-capacitor-resistor
low-Z	Low-impedance
PCB	Printed circuit board
RX	Receiver
RMS	Root mean square
SMD	Surface mounted devices
SNR	Signal-to-noise-ratio
TIA	Transimpedance-amplifier
TOSM	Through-open-short-match
TX	Transmitter
